# Local Reirradiation of Recurrent Non-small Cell Lung Carcinoma Resulting in Long Disease-free Survival, Although in the Presence of Osteonecrosis

**DOI:** 10.7759/cureus.3471

**Published:** 2018-10-22

**Authors:** Marloes Duijm, Bas Schipaanboord, Patrick V Granton, Joost Nuyttens

**Affiliations:** 1 Radiation Oncology, Erasmus Medical Center Cancer Institute, Rotterdam, NLD

**Keywords:** reirradiation, lung cancer, chest wall toxicity, osteonecrosis, disease-free survival

## Abstract

High-dose reirradiation of the thorax can be offered to patients with only local disease progression of non-small-cell lung cancer (NSCLC) resulting in promising disease-free-survival. However, much is still unknown about related side-effects and occasionally an uncommon presentation can be caused by reirradiation. In this case report, we present a patient with a 3.5-year progression-free survival, although in the presence of a late, unexpected toxicity. A dosimetric analysis was performed to investigate the possibility of radiation-induced toxicity.

## Introduction

Despite the continuous development of radiation techniques for patients with advanced lung cancer, local or locoregional failure does occur. A meta-analysis showed a 35% locoregional progression at five years after sequential chemoradiotherapy and 29% after concomitant chemoradiotherapy in locally advanced non-small-cell lung cancer (NSCLC) [1]. Following a course of curative intent radiotherapy, patients presenting with local failure and limited, non-progressive, metastatic disease can be considered for additional localized radiotherapy (i.e., reirradiation). However, the prescription dose of reirradiation is often limited by the radiation tolerance of the surrounding organs at risk (OAR). Since no clear OAR dose constraints in the case of reirradiation are known, radiation-oncologists base their prescription schedules on single-center studies [2-3]. So, in the case of reirradiation, caution should be taken with regard to the total dose delivered to the surrounding structures [4]. In this case report, we present a patient who was reirradiated and is until now 44 months free of disease; however, this is at the expense of some rarely seen toxicity.

## Case presentation

**Figure 1:**
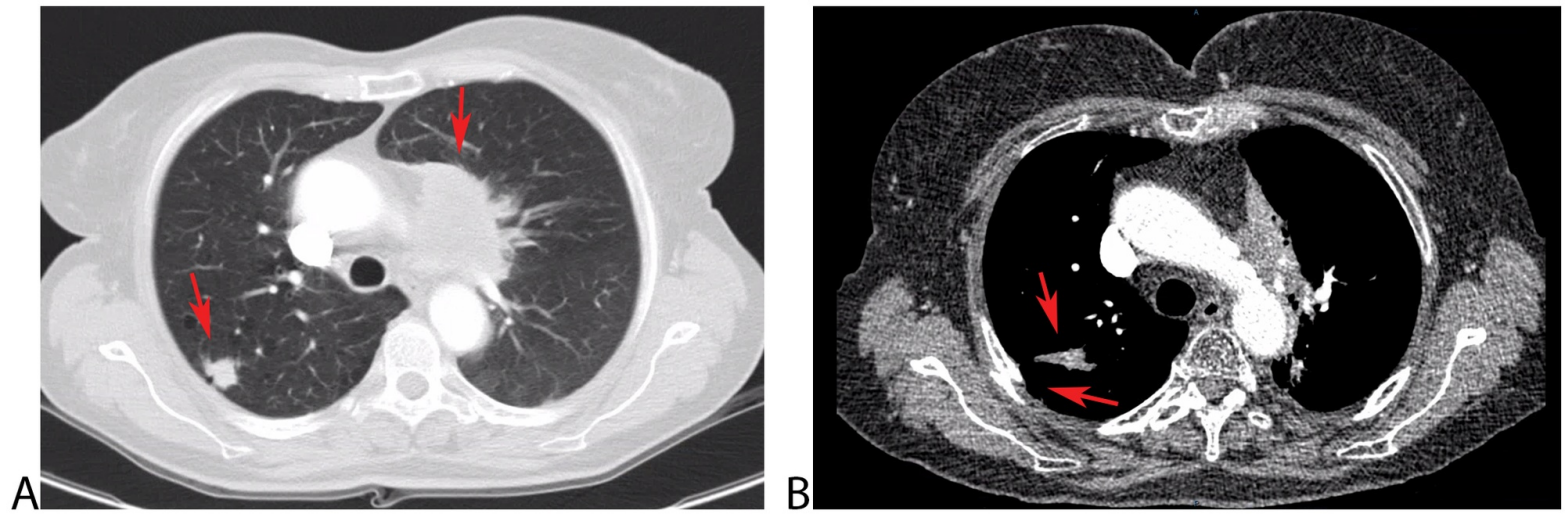
Computed tomography (CT) before and after first irradiation A) Transversal slice of both tumors on the CT scan 6.5 years ago at the time of diagnosis; large paramediastinal tumor on the left with some invasion in the aortopulmonary window (maximum size 65 mm) and second tumor on the dorsal side of the right upper lobe (maximum size 18 mm); B) CT of the thorax, transversal slice, two years after first irradiation. This scan shows a soft tissue component at the place of the right tumor and a fracture on the fifth right rib at the same level.

The patient was a 65-year-old Caucasian woman with a history of 25-pack-year cigarette use. She was diagnosed with double primary NSCLC, a centrally necrotizing tumor with invasion of the mediastinum in the left upper lobe (stage T_4_N_x_M_0_) and a peripheral tumor located dorsally in the right upper lobe (stage T_1_N_0_M_0_) (Figure 1A). Initial treatment consisted of four cycles of carboplatin and pemetrexed, followed by sequential radiotherapy to the left upper lobe (60 Gy in 20 fractions) and stereotactic body radiotherapy (SBRT) of the right upper lobe (51 Gy in three fractions, fiducial tracking).

Initially, she did well but developed a pathology-proven recurrence in the left upper lobe after 2.5 years. This local recurrence was an in-field recurrence and she was reirradiated with SBRT (50 Gy in five fractions, fiducial tracking). During that period, she also developed some first complaints of pain at her left thorax radiating to her left arm, for which she got a cervical epidural with corticosteroids followed by fentanyl, 12 mcg, with rescue medication consisting of paracetamol and diclofenac. This pain persisted over the years with some flares over time. The presence of a metastatic tumor as the cause of the complaints had been excluded by thoracic computed tomography (CT) scans and positron emission tomography (PET) imaging. Additionally, there were also some persistent complaints of pain on the right side of the thorax, which were most likely caused by a radiation-induced fracture of the ribs (Figure 1B).

At this moment, 6.5 years after her initial radiotherapy and without any additional therapy after reirradiation, the patient is still alive without the presence of disease. Unfortunately, she keeps having lingering complaints related to her left thorax. These complaints started five years after the first treatments when she was hospitalized with severe pain and restricted movements of her left arm. This was caused by a parasternal abscess due to osteonecrosis of the sternoclavicular joint, and she was treated with surgical drainage and antibiotics. During the following months, it was difficult to control the pain. Four months later, she was hospitalized again because of the persistent inflammation of the skin, located laterally of the sternum at the left thorax with infiltration to underlying structures. Treatment consisted of antibiotics and cleaning of the wound three times a day. The existence of an abscess was excluded with a CT scan, but echography of the thorax showed the presence of a tract. A biopsy showed a combination of an active and chronic inflammation.

**Figure 2:**
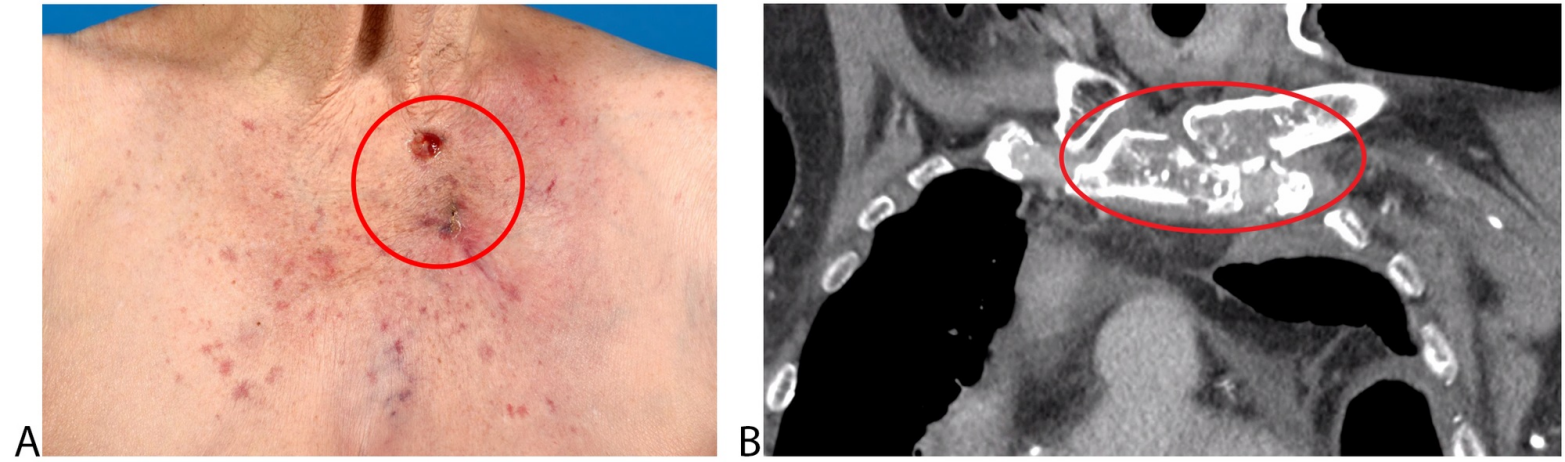
Ulcers based on osteonecrosis A) Physical exam of the two painful left parasternal ulcers; B) Coronal slice in a CT of the thorax made six years after initial radiotherapy showing destruction of the left sternoclavicular joint

Due to the persistent complaints, a plastic surgeon was consulted to discuss possible surgical interventions for the persistent ulcus based on osteonecrosis (Figure 2A)*. *The affected area was resected, followed by reconstruction with a mini deep inferior epigastric artery perforator (DIEP) flap.

**Figure 3:**
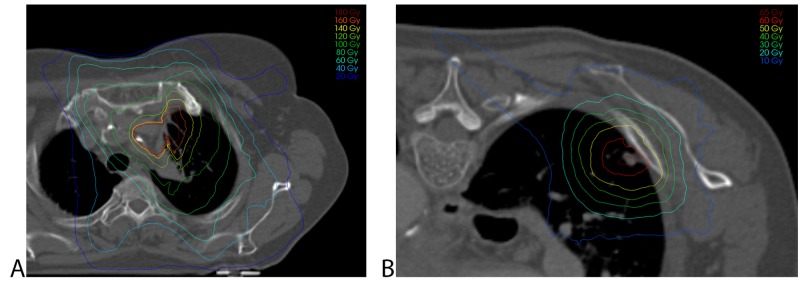
Dosimetric details of the irradiation A) Accumulated dose projected on the planning CT scan of the conventional planning, showing 100 - 140 Gy EQD_2_ (α/β-ratio of 3 Gy) in the sternoclavicular joint; B) Planning CT scan for stereotactic radiotherapy to the right upper lobe in absolute dose. Maximum dose in the fifth rib is 306 Gy EQD_2_ (α/β-ratio of 3 Gy). EQD: equivalent dose

To determine whether the radiotherapy contributed to the osteonecrosis of the sternoclavicular joint (Figure 2B), an analysis of the radiation schedules was performed. In order to analyze the total dose delivered to the affected structures, we accumulated the doses of the three radiotherapy treatments. Since the fractionation schemes were different between treatments, doses were converted to an equivalent dose of 2 Gy (EQD_2_) before accumulation. An α/β-ratio of 3 Gy was used for all structures because our main point of concern was the late effects in the organs at risk. Dose accumulation was performed by registering the CT scans and subsequently warping the dose according to the calculated spatial transformation. For this, the scans were initially aligned using a rigid registration followed by a deformable registration. The accumulated dose showed a maximum dose between 100 - 140 Gy EQD_2_ in the sternoclavicular joint (Figure 3A). Since the rib fracture was diagnosed before reirradiation, the planning CT scan could be used for dose analysis. The maximum point dose (D_max_) in the fifth right rib was 306 Gy EQD_2_, based on the dose volume histogram (Figure 3B). 

## Discussion

Whereas reirradiation of the thorax has traditionally been reserved for palliation in the setting of disease recurrence, nowadays its role is becoming more and more curative. By giving a highly precise adaptive dose, disease-free survival can be improved. Median disease-free survival of 14 high-dose treatment studies was 10 months with a mean overall survival of 17.7 months [4]. The disease-free survival in our case is 44 months, where recent imaging of the thorax showed no recurrence of the disease.

Unfortunately, our patient was having complaints of pain in combination with the presence of osteonecrosis. Because of her history with radiation, radiation-induced toxicity is suspected to be the cause of the osteonecrosis, which has resulted in abscess and pain. The CT scan clearly showed a destruction of the sternoclavicular joint (Figure 2B)* *and the accumulated radiation dose in the joint was between 100 - 140 Gy EQD_2_. Based on these findings and the absence of alternative explanations, local toxicity from radiotherapy is a likely explanation for the patient’s presentation.

Literature of dosimetric parameters related to toxicity after reirradiation is scarce. Various studies report the maximum dose to organs at risk at the time of reirradiation, but often an analysis of the accumulated dose to organs at risk is missing. Thus, severe toxicity reported after reirradiation is mostly located in the lung, esophagus, or great vessels; Grade 3-4 lung toxic effect occurred in 10% of patients, 2% showed Grade 3 esophagitis, and 3% died of a bleeding related to the treatment [4].

When focusing on skin and bone toxicity, mostly rib fractures and/or pain is reported. Only a few cases of ulceration and/or necrosis can be found. Hoppe et al. [5] described one patient with Grade 4 skin necrosis from reirradiation with SBRT. No specific details of the planning were provided, but we can derive that three beams within a narrow angular range were used because of lung irradiation of a contralateral NSCLC four years earlier. The D_max­_ of the SBRT plan approached 90% of the prescription dose (187 Gy EQD_2_, α/β-ratio of 3 Gy). In another study, two cases of Grade 3 chest wall ulcers were described after reirradiation with SBRT, whereas the initial treatment consisted of external beam radiotherapy [6]. The dose to the skin of those cases was not described; however, dose-constraints for the skin were D_1 cc_ of ≤ 40 Gy and D_10 cc_ ≤ 35 Gy (D_x cc_ was defined as the dose in x cc of the organ at risk). The dose was given in four fractions, which equals 104 Gy EQD_2_ and 83 Gy EQD_2_ using α/β-ratio of 3 Gy, respectively. A case report on post-SBRT described lung necrosis with abscess and fistulation of the chest wall (CW) [7]. Toxicity occurred 17 months after one radiation course, and analysis of the treatment plan showed a CW V_30 Gy_ of > 66 cc and skin D_max_ of 34 Gy (V_x Gy_ is defined as the volume of the organ at risk receiving x Gy). The dose was delivered in five fractions, which equals V_54 Gy, EQD2_ and 67 Gy EQD_­2_ using α/β-ratio of 3 Gy. Additionally, this patient received four cycles of carboplatin/paclitaxel six months after SBRT because of new lung nodules.

The limited amount of data reporting similar cases like ours makes it hard to compare our data. The hypothesis that our patient, although unclear by which factors, was more sensitive to toxicity can be supported with the analysis of the fractured rib which occurred after the first radiation period. Several studies have described V_30Gy_ as a prognostic factor for chest wall toxicity and all concluded that a CW V_30 Gy_ < 30 cc, or sometimes even bigger, should be safe [8-9]. In our case, the CW V_30 Gy_ was lower than 20 cc, which corresponds to a 9% risk on Grade 1-2 CW toxicity [10]. Predictors for rib fracture in the study by Park et al. were rib D_4.6 cc_ > 140 Gy EQD_2_ and rib V_160 Gy, EQD2_ > 3.2 cc [11]. Therefore, according to that study, the chance of developing a rib fracture in our patient was low, having D_4.6 cc_ of 76 Gy EQD_­2_ and V_160 Gy, EQD2_ of 2.9 cc.

With regards to our study, there are some limitations concerning dose accumulation from subsequent radiation treatments separated by a long time interval (i.e., > 1 year). First, it is important to note that dose accumulation does not transfer the actual beams and their corresponding dose deposition but rather accumulates the dose delivered to each individual volume into one dose distribution, which assumes that the two volumes at different time points reflect the same tissue. Additionally, in all planning CT scans, the patient was positioned differently; the conventional radiotherapy was given in a supine position with both arms up, the SBRT of the right tumor in the prone position, and the reirradiation in a supine position with the left arm down (because of the pain the patient was having). The gross difference in anatomy, because of the different positions, impedes the accuracy of the registration; nevertheless, the accumulated dose distribution provides a rough estimation of the dose delivered to the various structures.

## Conclusions

Reirradiation of the thorax with a curative dose can result in prolonged disease-free-survival in cases of advanced NSCLC. However, some unexpected cases of toxicity are occasionally observed which can highly influence the daily life of the patient. Promising results have been achieved with high-dose reirradiation; however, more toxicity and/or dose-constraint-related research is necessary for better toxicity prediction and evidence-based decision making.
